# Using Leaf Temperature to Improve Simulation of Heat and Drought Stresses in a Biophysical Model

**DOI:** 10.3390/plants9010008

**Published:** 2019-12-19

**Authors:** Ruchika S. Perera, Brendan R. Cullen, Richard J. Eckard

**Affiliations:** Faculty of Veterinary and Agricultural Sciences, University of Melbourne, Melbourne, VIC 3010, Australia; bcullen@unimelb.edu.au (B.R.C.); rjeckard@unimelb.edu.au (R.J.E.)

**Keywords:** energy budget, leaf temperature, air temperature, simulation modelling, heat stress recovery, DairyMod, pasture

## Abstract

Despite evidence that leaf temperatures can differ by several degrees from the air, crop simulation models are generally parameterised with air temperatures. Leaf energy budget is a process-based approach that can be used to link climate and physiological processes of plants, but this approach has rarely been used in crop modelling studies. In this study, a controlled environment experiment was used to validate the use of the leaf energy budget approach to calculate leaf temperature for perennial pasture species, and a modelling approach was developed utilising leaf temperature instead of air temperature to achieve a better representation of heat stress impacts on pasture growth in a biophysical model. The controlled environment experiment assessed the impact of two combined seven-day heat (control = 25/15 °C, day/night, moderate = 30/20 °C, day/night, and severe = 35/25 °C, day/night) and drought stresses (with seven-day recovery period between stress periods) on perennial ryegrass (*Lolium perenne* L.), cocksfoot (*Dactylis glomerata* L.), tall fescue (*Festuca arundinacea* Schreb.) and chicory (*Cichorium intybus* L.). The leaf temperature of each species was modelled by using leaf energy budget equation and validated with measured data. All species showed limited homeothermy with the slope of 0.88 (*P* < 0.05) suggesting that pasture plants can buffer temperature variations in their growing environment. The DairyMod biophysical model was used to simulate photosynthesis during each treatment, using both air and leaf temperatures, and the patterns were compared with measured data using a response ratio (effect size compared to the well-watered control). The effect size of moderate heat and well-watered treatment was very similar to the measured values (~0.65) when simulated using T leaf, while T air overestimated the consecutive heat stress impacts (0.4 and 0). These results were used to test the heat stress recovery function (Tsum) of perennial ryegrass in DairyMod, finding that recovery after heat stress was well reproduced when parameterized with T sum = 20, while T sum = 50 simulated a long lag phase. Long term pasture growth rate simulations under irrigated conditions in south eastern Australia using leaf temperatures predicted 6–34% and 14–126% higher pasture growth rates, respectively at Ellinbank and Dookie, during late spring and summer months compared to the simulations using air temperatures. This study demonstrated that the simulation of consecutive heat and/or drought stress impacts on pasture production, using DairyMod, can be improved by using leaf temperatures instead of air temperature.

## 1. Introduction

Climate change projections for Australia indicate increasing frequency and magnitude of extreme climate events such as heat waves, droughts, extreme precipitation and frost occurrences in the coming decades [1], which are likely to reduce productivity and profitability of pasture-based systems [2]. A recent study conducted in southeastern (SE) Australia showed that the anticipated changes to the pasture growth patterns under future climate change reported by [3,4] are already occurring under current climate change, including increased pasture yield variability over the major growing seasons (autumn and spring) and a decreased spring season growth leading to shorter growing season lengths [5]. These changes were more prominent in the most recent period (2002–2015) compared to the periods before and were mainly caused by the increased occurrences of heat and drought stress [5]. 

The optimal temperature range for growth of temperate pasture species lies between 15 °C and 23 °C [6,7]. Beyond this range, growth and physiological processes decrease in plants. Net photosynthesis reduction of perennial ryegrass (*Lolium perenne*) and Kentucky blue grass (*Poa pratensis)* starts at temperatures above 25 °C [8,9]. For cocksfoot (*Dactylis glomerata)*, the optimum temperature range for maximum net canopy photosynthesis is between 19 and 22 °C and the values declining to lowest at 31 °C [10]. Physiological impairments due heat stress occurs in plants mainly due to reduction of Rubisco activity [11,12], reduction of maximum photochemical efficiency of photosystem II [13,14], reduction of apparent electron transport rate of photosystem I [15], production of reactive oxygen species (ROS) [16,17] and subsequent damages to the cell membranes [18]. Since high temperature stress often coincides with moisture limitation under field conditions, the combined impacts could be over and above the effects of individual stresses [13,14,19].

Accurate assessment of the effects of heat and drought stresses on crop processes is important to identify correct management and adaptation strategies in order to stabilize production. Many crop simulation models incorporate heat and drought stress impacts on growth and developmental processes. For example, the Agricultural Production Systems sIMulator (APSIM) simulates leaf senescence of wheat (*Triticum aestivum* L.) due to heat stress between 32 °C and 34 °C (daily maximum temperatures) [20,21]. Ecosys simulates heat stress impacts on seed set of wheat above 33 °C during anthesis and post anthesis periods [22]. Likewise, grain filling in wheat crop stops when the maximum temperature exceeds 38 °C in STICS (Simulateur mulTIdisciplinaire pour les Cultures Standard) model [23]. However, many of the biophysical models are parameterized with air temperatures taken from the meteorological data assuming that air temperature is a fair representation of the environment at which crops are grown. It is well known that leaf temperature can differ from air temperatures [24], depending on the structural and physiological characteristics of the leaf. Under well-watered conditions, it has been observed that plant leaves are generally cooler than air at above optimum temperatures and hotter than air at below optimum temperatures [25,26,27]. This phenomenon is called “limited homeothermy” and considered as an adaptive response of plants to maintain leaf temperatures within the optimum range for photosynthesis [25].

Ignoring this leaf-to-air temperature difference may cause uncertainties in assessing heat and drought stress impacts by crop simulation models [28]. It may also result in parameterizing heat stress functions with unrealistic temperature threshold values [28,29] and in turn predicting unrealistic results [30]. Use of leaf/canopy temperatures would reduce such uncertainties and improve the simulations. For example, canopy temperature measured during the anthesis of the rye canopy (*Secale cereal* L.) was 2 °C cooler than the air when irrigated and it was 7 °C warmer than the air under rainfed conditions [28]. Based on this observation, heat stress effects on rye grain number observed in controlled experimental conditions were able to reproduce well under field conditions when only the stress thermal time ((STT) temperature sums accumulated above high temperature stress threshold during the heat sensitive growth stage of the crop [31]) was calculated using canopy temperatures, but not using air temperatures. Similarly, the use of canopy temperature calculated using energy balance slightly improved heat stress effects of wheat than using air temperature in wheat models [29]. 

Leaf energy balance provides a process-based approach to incorporate canopy temperature effects into crop simulation models [24,25,29]. In an energy balance approach, the summation of net radiation (absorbed–emitted), latent heat flux (energy required to evaporate water) and sensible heat flux (energy required to warm or cool the leaf) at the leaf surface should be equal to zero. The latent heat is proportional to the transpiration rate and the sum of boundary layer and stomatal conductance to water, while sensible heat flux is proportional to the difference between leaf and air temperature and boundary layer conductance to heat. Therefore, leaf temperature can be derived from the sensible heat flux term. Using these thermodynamic principles and linearization techniques used by Penman [32], Campbell and Norman [24] derived an equation to determine leaf temperature in a straightforward way, using air temperature, wind speed, radiation and vapor pressure deficit, which enables the calculation of the leaf temperature using the recorded weather data and crop specific parameters. 

DairyMod is a mechanistic biophysical pasture model developed to predict grazing dynamics across a range of climates, soil types, forage species and management under conditions in Australian and New Zealand [33]. In the DairyMod model, temperature response to photosynthesis describes the growth of pasture species using minimum and optimum temperatures where growth limitation occurs at temperatures above and below the optimal temperatures. Additionally, growth restrictions under high temperatures are simulated in the model using an empirical function referred to as the high-temperature stress coefficient, which is a scale ranging from 0 (full stress) to 1 (no stress). The model reduces photosynthesis and subsequent growth if the maximum daily temperature exceeds the high temperature onset and approaches maximum when it reaches full stress [34]. High temperature stress thresholds (onset and full) for perennial ryegrass have been parameterized using experimental evidence [35]; however, there is little data available to parameterize the recovery from heat stress (T sum) in the model. 

The broad objective of this study was to evaluate whether the use of leaf temperature compared to air temperature can better simulate the impact and recovery of consecutive heat and drought stresses leading to improved prediction of heat and drought impacts in biophysical models. The specific objectives were: (1) to test the limited homeothermy hypothesis for four temperate pasture species commonly grown in SE Australia; (2) to validate the leaf energy budget equation using leaf temperature data measured under well-watered (WW) and water-stressed (WS) conditions at three temperature levels; (3) to parameterize the high temperature stress recovery function in DairyMod, and finally, (4) to model leaf temperature using the leaf energy budget equation and use these in simulations in DairyMod to estimate the uncertainty associated with use of air temperature to predict pasture growth at two sites in SE Australia.

## 2. Results

### 2.1. Test for the Limited Homeothermy of Pastures

**Figure 1:**
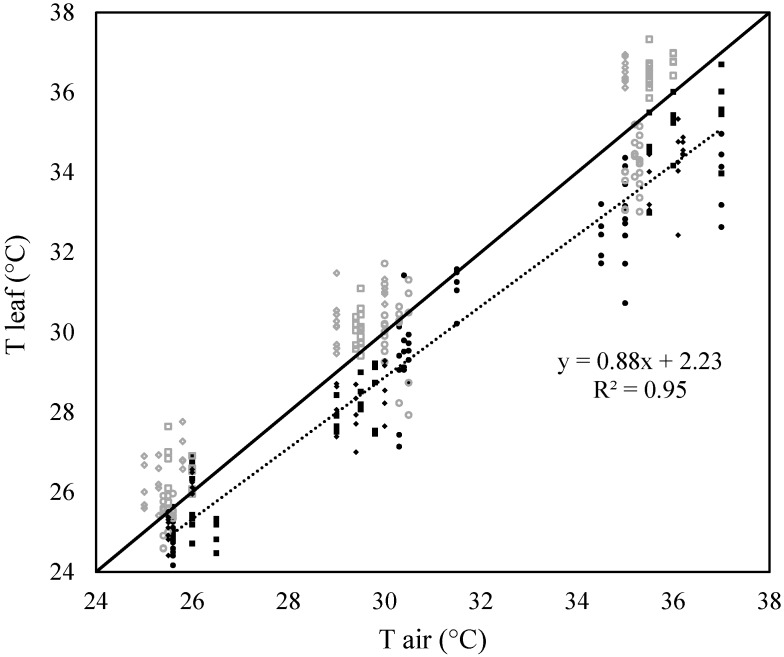
Relationship between air temperatures inside the growth chambers and leaf temperatures (measured using infra-red thermal images) of perennial ryegrass, cocksfoot, tall fescue and chicory at three temperatures (25 °C, 30 °C and 35 °C) and two watering levels during day 2(●), 4(■) and 7(♦) of the second combined heat and drought stress treatment. Filled black symbols represent well-watered plants and open grey symbols represent water-stressed plants. The thick line refers to the 1:1 reference line and dotted line represents regression line for well-watered plants. n = 294.

The slope of the relationship between leaf and air temperature for irrigated plants was 0.88 (R^2^ = 0.95). This slope was significantly less than 1 (P = 0.001) and greater than 0 (*P* < 0.001) (Figure 1), therefore the pasture species used in this study would be classified as limited homeotherms, with the ability to buffer leaf temperature against the variation in the ambient air temperature. 

In general, leaf temperatures were cooler than air temperatures under well-watered (WW) conditions, while leaves were warmer than air temperatures under water-stressed (WS) conditions (Figure 1). The difference between WW and WS plants at each temperature on each day was significant at *P* = 0.05 level, except for the moderate heat stress on day 2. The difference between WW and WS plants was smaller on day 2 of the stress treatment and it increased as the combined heat and drought stresses progressed through time. For instance, the difference between WW and WS plants on day 2 was 1.1 °C under severe heat stress (35 °C), but increased to 2.3 °C on day 7 (*P* < 0.001).

### 2.2. Ability of the Leaf Energy Budget Equation to Model Leaf Temperature 

**Figure 2:**
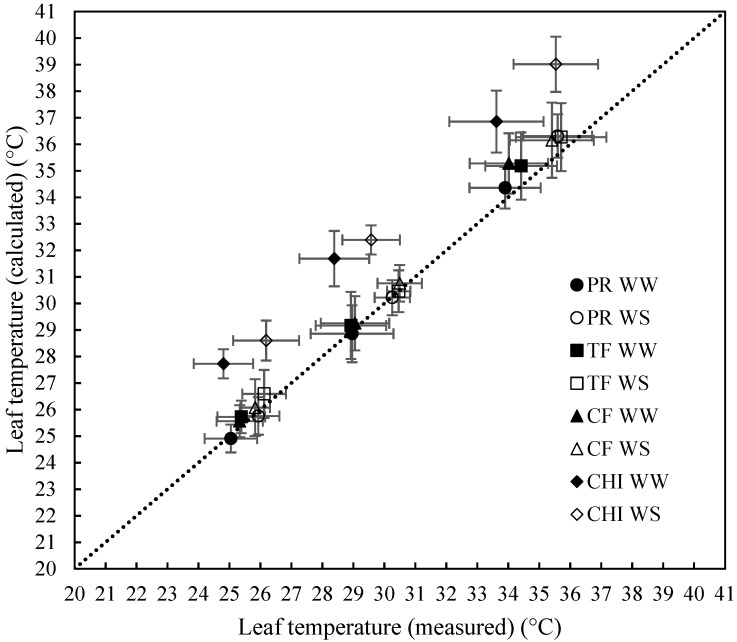
Measured (using infra-red camera) and modelled (using leaf energy budget equation) leaf temperature values of perennial rye grass (PR), cocksfoot (CF), tall fescue (TF) and chicory (CHI) at three temperature (25 °C, 30 °C and 35 °C) and two watering levels (well-watered (WW) - filled symbols, water-stressed (WS) - open symbols) during the second combined heat and WS treatment. Means (3–5 plants) of each species are presented with error bars representing ± standard deviation. The dotted black line is the 1:1 reference line.

The measured leaf temperature is compared with modelled temperatures using the leaf energy budget for each pasture species in Figure 2 with summary statistics presented in Table 1. The mean bias indicates that there is an overprediction of 0.98 °C on average for all the data (Table 1). Chicory showed the highest mean bias of 3.06 °C while grasses had mean bias less than 0.5 °C. Mean prediction error was also less than 5% for grasses indicating excellent model prediction but was 10.8% for chicory. Similarly, modelling efficiency was above 0.9 for the grass species, while for chicory it was 0.37. Bias correction factor (C_b_) and Variance Ratio (V) were above 0.9 for all the categories, indicating that there were only small deviations from 1:1 reference line and that variance in measured and modelled data were similar in all the categories. In general, the leaf energy budget equation predicted the leaf temperature more accurately for grasses (with higher R^2^, Pearson’s correlation coefficient (r), modelling efficiency (MEF), concordance correlation coefficient (CCC) and lower mean prediction error (MPE)) than chicory.

### 2.3. Use of T Leaf and T Air to Simulate Photosynthesis 

**Figure 3:**
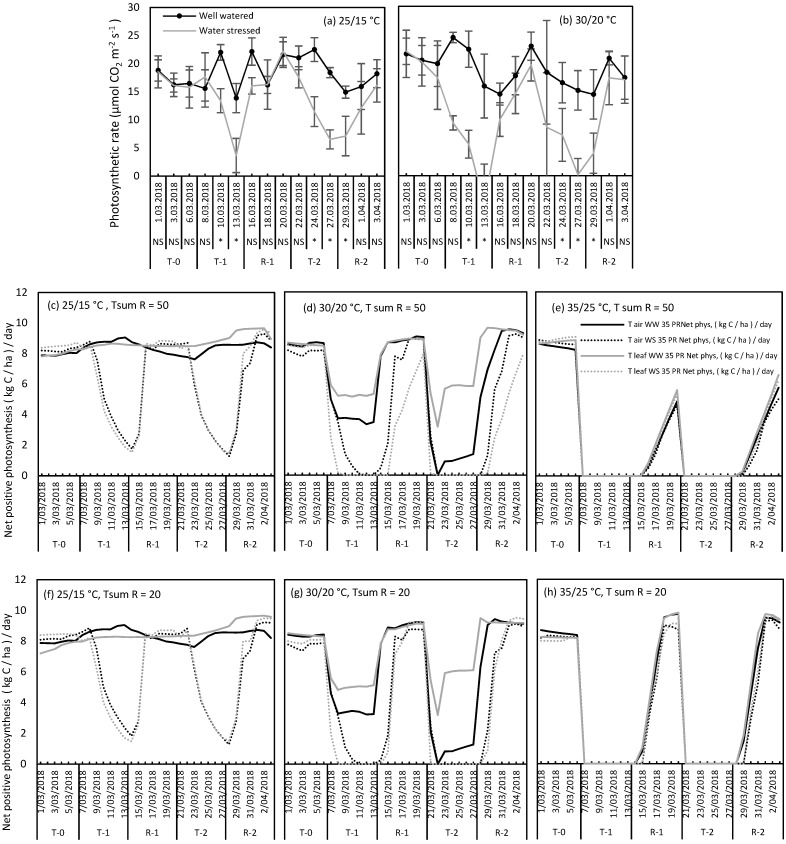
Net photosynthetic rates (µmol CO_2_ m^−2^ s^−1^) measured under control and moderate heat stress (**a**,**b**) and net positive photosynthesis ((kg C / ha ) / day) modelled using DairyMod biophysical model (**c–h**). Middle graphs (**c–e**) represent modelled data with T sum = 50 and the bottom graphs (**f–h**) represent modelled data with T sum = 20. In graphs a and b, black line indicates WW and grey lines indicate WS plants with significant differences between WW and WS at each day is shown in asterisk marks. NS = Not Significant. In Figure c–h, black lines represent simulations run using air temperatures and grey lines represent simulations run using leaf temperatures. Thick lines denote well-watered plants while dotted lines denote water-stressed plants. T-0 represents pre-treatment period, T-1 and T-2 represent 7-day treatments and R-1 and R-2 represent 7-day recovery periods.

Perennial ryegrass showed a significant decrease in the measured leaf photosynthesis rates at the end of consecutive WS treatments by 74% and 65%, respectively, compared to the WW plants at the control temperature (25 °C) (Figure 3a). Heat stress at 30 °C only decreased leaf photosynthetic rates by 35% and 27% during consecutive treatments (Figure 3b). In contrast, photosynthetic rates reached zero (100% reduction) when the heat (30 °C) and drought stresses were imposed together (Figure 3b). At both temperatures, perennial ryegrass fully recovered from combined heat and WS treatments at the end of each recovery phase.

DairyMod simulated photosynthetic rates of perennial ryegrass using T-air versus T-leaf and Tsum 50 versus Tsum 20 (Figure 3c–h) followed a similar pattern to the measured photosynthesis data (Figure 3a,b). Correlation coefficients showed that there was a strong (r > 0.85) (*P* < 0.05) correlation between measured and modelled data in WS treatments, while no such significant pattern was observed in WW plants (Table 2). Recovery of photosynthesis after heat stress was reproduced well when the DairyMod model was parameterised using Tsum = 20, while there was a long lag phase in the recovery period at combined severe heat and drought treatments when Tsum 50 was used. For instance, photosynthesis rates recovered only by 50% (Figure 3e) when Tsum 50 was used irrespective of the leaf temperature or air temperature used. Even though no photosynthetic data were available at 35 °C, other physiological data such as maximum photochemical efficiency of photosystem II, leaf elongation rates and relative water contents measured in this same experiment provide evidence that perennial ryegrass fully recovered from severe heat and drought stress at the end of the seven-day recovery period [14], which was well simulated when Tsum 20 was used (Figure 3h).

**Figure 4:**
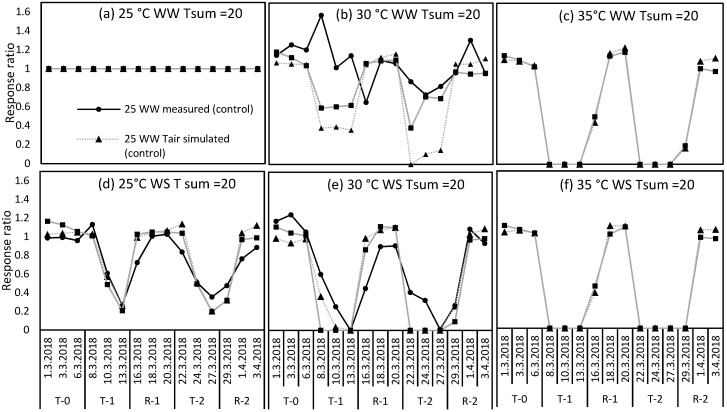
Response ratios (effect size compared to well-watered control) of perennial ryegrass for well-watered (WW, **a**,**b**,**c**) and water-stressed plants (WS, **d**,**e**,**f**) at three temperature levels (25 °C, 30 °C, 35 °C) calculated using photosynthesis rates of measured data (●) and simulated data using air temperature (▲) and leaf temperature (■) during the experiment. T-0 is the pretreatment period, T-1 and T-2 are treatments and R-1 and R-2 are recovery periods.

Effect size (calculated as response ratio) of each temperature and watering treatment compared to the WW control was shown for measured and simulated data (using Tsum 20) in Figure 4. Response ratios calculated for measured data were well reproduced by both DairyMod simulations (using air temperature and leaf temperature) in WS plants at control and moderate temperatures. However, simulations with leaf temperature performed better than air temperature in simulating the effect size of WW plants under moderate heat stress (Figure 4b). For instance, response ratio calculated using leaf temperature simulations was reduced from 1 to 0.6 during the first treatment period (T-1) which was very similar to the actual reduction of response ratio (from 1 to 0.65) in the measured data, but it was reduced to 0.37 in the simulations with air temperature. During the second treatment, reduction in response ratio calculated with simulations run with leaf temperature decreased to 0.38 on day 2 of the treatment but recovered quickly to 0.7 on day 4, which is again similar to the measured reduction (0.7). In contrast, response ratios calculated using simulations run with air temperature reached minimum values of zero. Likewise, at both consecutive moderate heat stress treatments under WW conditions, DairyMod simulations with air temperature overestimated the actual impacts of moderate heat stress on perennial ryegrass. This was also confirmed by the evaluation statistics shown in Table 3, where under moderate heat stress and WW treatment (Figure 4b), RMSE was larger (0.54) for simulations run with air temperature, while it was much smaller (0.36) for simulations run with leaf temperature. Likewise, MAE was also higher for air temperature (0.42) and lower for leaf temperature (0.25) (Table 3). 

### 2.4. Uncertainty in Perennial Ryegrass Growth at Ellinbank and Dookie when Using Air Temperature in the Simulations

**Figure 5:**
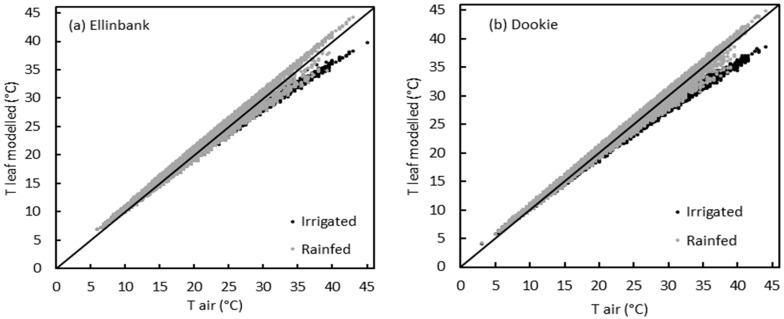
Relationship between air temperature and modelled perennial ryegrass leaf temperature at Ellinbank (**a**) and Dookie (**b**) under irrigated (black) and rainfed (grey) conditions. Black line = 1:1 reference line.

When applying a leaf energy budget to calculate leaf temperature, the modelled leaf temperatures of perennial ryegrass under irrigated conditions were generally cooler than air at both Ellinbank and Dookie sites above maximum air temperatures of 18 °C and 16 °C, respectively (Figure 5). Below these temperatures, modelled leaf temperatures were warmer than air agreeing with the limited homeothermy hypothesis as tested with the measured data. Modelled leaf temperatures scattered more widely around the 1:1 reference line at both sites under rainfed situations. This was due to the presence of both wet and dry days within a year where leaf temperatures are cooler when there is enough soil moisture to transpire, while leaf temperatures tend to be warmer when there is limited soil moisture. 

**Figure 6:**
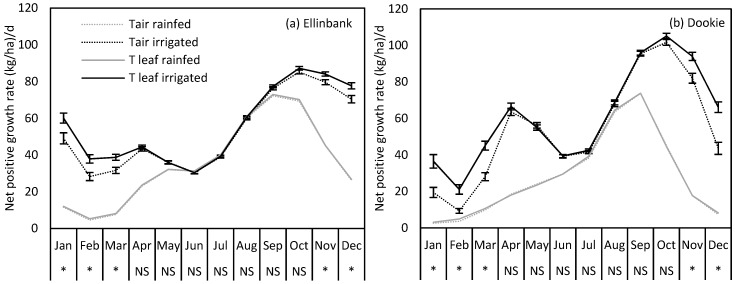
Simulated pasture growth rates (net positive growth rate, kg DM/ha.day) of perennial ryegrass under irrigated (black) and rainfed (grey) conditions at (**a**) Ellinbank and (**b**) Dookie. Simulations using air temperature are shown with dotted lines while those with leaf temperature are shown with thick lines. Mean pasture growth rates for each month (1960–2015) are shown with error bars representing ± standard errors. Significant differences (*P* = 0.05) between air temperature and leaf temperature in each month for irrigated simulations are shown in the X axis using (*). NS = not significant.

DairyMod simulated no significant yield difference between using leaf temperature and air temperature under rainfed situations at either site. However, when pasture paddocks were irrigated, there was a significant (*P* < 0.05) increase in predicted pasture growth rates simulated using leaf temperature from November through to March at both sites, compared to air temperature. For instance, simulated perennial ryegrass production at Ellinbank in Nov, Dec, Jan, Feb and Mar increased by 6%, 10%, 22%, 34%, and 23%, respectively, while at Dookie the simulated pasture production increased by 14%, 52%, 88%, 126% and 60%, respectively, when simulated using leaf temperature compared to air temperature (Figure 6).

## 3. Discussion 

This study demonstrated that the impacts of heat and water stresses on perennial pasture plants could be better simulated in a biophysical model using leaf temperature, rather than air temperature, because it captures the interactions between air temperature and water status of the plant. The four pasture species used in this study showed limited homeothermy under irrigated conditions indicating that pastures can buffer temperature variations in their surrounding environment through transpirational cooling. Leaf temperature values modelled with a leaf energy budget equation were in good agreement with the measured data for grasses as indicated by higher modelling efficiency (~0.95) and lower mean prediction error (~3%). Leaf temperatures better simulated the effects of moderate heat stress on photosynthetic rates of perennial ryegrass while simulations with air temperatures overestimated the impacts. The pattern of photosynthesis recovery after heat stress was well reproduced by DairyMod when Tsum = 20 was used while T sum = 50 simulated longer lag phase between stress and full recovery. When the modelled leaf temperature was used, both Dookie and Ellinbank sites simulated under irrigated conditions predicted higher pasture growth rates in late spring and summer periods compared to the simulations run with air temperatures. These results confirmed that uncertainty in simulating heat and drought stress on pasture growth in DairyMod can be reduced by using leaf temperature in the simulations and parameterizing high temperature stress recovery function with T sum = 20.

The slope of WW plants (considering all pasture species) (Figure 1) was 0.88, which was significantly less than 1. Using leaf temperatures from 62 species measured at an air temperature gradient of 50 °C, a slope of 0.67 was reported in a previous study [25]. The slope of the grass species observed in this study was greater than that observed by [25], hence the difference was small in the leaf-to-air temperature. This is because the grasses have narrow leaves and smaller leaf characteristic dimensions compared to broad leaves. According to the energy balance equation, narrow leaves have a greater convective energy exchange rate compared to the broader leaves, hence grass leaves maintain temperature nearer to air temperature [24]. 

Well-watered plants often maintained cooler canopies than air through transpirational cooling (Figure 1) at all temperature levels. Photosynthesis and respiration enzymes in plants have a narrow thermal tolerance range. Therefore, cool canopies help plants to remain physiologically active in the periods of high air temperatures [13,25]. Transpirational cooling may also help to reduce the temperature at the crown (plant–soil interface) [36]. During vegetative growth, apical meristems are at the crown level [37,38,39] and they produce phytomers, which are the repeating units of vegetative growth [40]. Supra optimal temperatures at the crown area could damage the apical meristems and in turn challenge plant survival [41]. Therefore, transpirational cooling helps plants to maintain growth and physiological functions as well as plant survival. 

**Figure 7:**
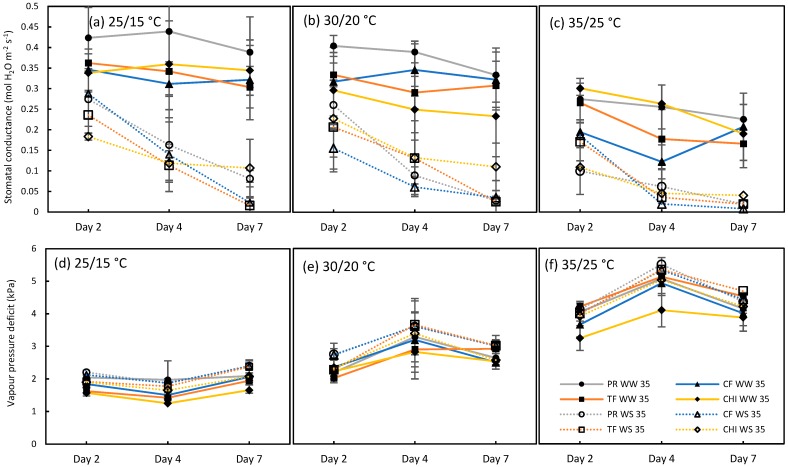
Stomatal conductance (**a**–**c**) and leaf to air vapour pressure deficits (**d**–**f**) of perennial ryegrass (PR), cocksfoot (CF), tall fescue (TF) and chicory (CHI) at control (25/15 °C, day/night), moderate heat stress (30/20 °C, day/night) and severe heat stress (35/25 °C, day/night) respectively during the day 2, 4 and 7 of the second heat and drought treatment. Filled symbols and thick lines represent WW plants and the open symbols and dotted lines represent WS plants. Mean (*n* = 3–5) is provided with the error bars representing standard deviation.

Compared to the WW plants, WS plants had warmer leaves at all temperature levels. This was mainly due to the gradual decrease in stomatal conductance and development of greater leaf to air vapor pressure deficits with time as the combined stresses progressed (Figure 7). This result is consistent with a previous study conducted with wheat under WW and WS conditions where the warmer canopies occurred under WS due to decreased transpiration rates associated with lower stomatal conductance [42]. 

Leaf temperatures calculated using leaf energy budget equation were in good agreement with measured values for grasses but, the equation did not work well for chicory. Chicory, being a dicot plant has stomata only in one side of the leaf (hypostomatic dicot) and in contrast, grasses possess stomata on both sides (amphistomatic monocots) [43]. Grasses show greater conductance for vapor than chicory because conductance occurs from both sides of the leaves in grasses. Lower vapor conductance in chicory might cause accumulation of incoming radiation loads inside the leaf leading to more over prediction (mean bias > 3 °C) compared to grasses (mean bias < 0.5 °C) (Figure 2). The slight but consistent over prediction observed for other grass species could be due to mutual shading experience by the surrounding leaves. Since the equation does not simulate this effect, the calculated leaf temperatures could be slightly higher than the measured values. 

**Figure 8:**
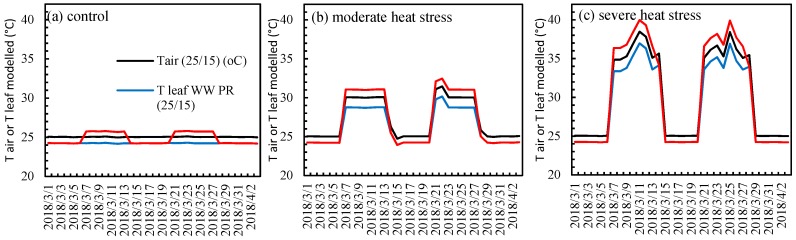
Maximum air temperatures (black line) and leaf temperatures of perennial ryegrass under well-watered (blue line) and water-stressed conditions (red line) on each day during the experiment at (**a**) control, (**b**) moderate heat stress and (**c**) severe heat stress treatments.

The difference between air and leaf temperature in plants has been well known for many years [26,44,45]; however, this relationship has not been used in the crop simulation models until recently [29]. Incorporation of leaf temperature in DairyMod simulations showed that even a small leaf to air temperature difference can cause a substantial impact when the temperatures are near to the upper end of supraoptimal temperature tolerance of a pasture species. For example, the leaf temperatures of WW perennial ryegrass under moderate heat stress was 1.3 °C cooler than air (Figure 8). This temperature difference prevented the perennial ryegrass leaves reaching the threshold temperature for heat stress impacts in DairyMod (30 °C) in the moderate heat stress treatment. However, DairyMod simulations that used air temperature started to simulate high temperature stress because air temperature reached the onset of high temperature stress threshold. When comparing the effect size on photosynthesis using response ratio, it was lower in the leaf temperature simulation which was in a good agreement with the measured data as indicated by the low RMSE and MAE than air temperature. 

The use of leaf temperature in simulations is increasingly important for regions like south eastern Australia, where maximum temperature during the late spring and summer months is likely to pass the onset of heat stress threshold of perennial ryegrass (30 °C) on some days, while soil moisture is still available for plant growth. Under such situations, transpirational cooling is likely to reduce leaf temperature and use of leaf temperatures would realistically simulate the high temperature response of pastures. Use of leaf temperature is equally important for simulating heat stress impacts of other crops. For example, in wheat, grain sterility due to heat stress occurs at 31 °C [46,47], but there is no impact at 30 °C. At 30 °C, a 1 °C increase in canopy temperature due decreased transpirational cooling associated with soil dryness can cause grain sterility. In contrast, at 31 °C, canopy temperature drops by 1 °C due to transpirational cooling can eliminate the impact of heat stress on grain sterility [29]. In this way, large over or underestimation errors in simulating grain yields are likely to occur when the leaf temperatures are ignored in the simulation. Further, high yielding wheat genotypes have been found to have cooler canopies associated with effective water uptake from the deep soil profile [48,49]. Similarly among temperate pastures, tall fescue and chicory have shown lower crown temperatures than perennial ryegrass under supra-optimal temperatures after cutting at different stubble heights [36], and the authors hypothesize that this cooler canopy would partly explain why tall fescue and chicory outperform perennial ryegrass in hot summers. 

The approach of using soil moisture stress index (GLF_water) to allocate stomatal conductance on each day for the energy budget developed in this study is similar to other studies that attempted to integrate canopy temperatures into crop models [29]. For example, in SIMPLACE<Lintul2> and SIMPLACE<Lintul5> models, soil water stress index was used to interpolate canopy temperatures between the high (no transpiration) and low (full transpiration) limits. In APSIM wheat, canopy temperatures were considered up to 6 °C warmer and 6 °C cooler than air under water-stressed and well-watered conditions, respectively, with canopy temperature change between those limits computed according to the relationship between canopy to air temperature difference and the ratio between actual and potential evapotranspiration [44,50]. However, in approach used in this study, the model has to be run using air temperature first to get the GLF_W data as this information is not available directly without running the model. Stomatal conductance values were then allocated to GLF_W value in each day to estimate the leaf temperature on each day. 

Based on the results, it is confirmed that the DairyMod model default values of high temperature stress recovery function (T sum = 100) for perennial ryegrass was too high because it takes more days to recover from high temperature stress than observed values [14]. Both values of T sum = 50 and T sum = 20 tested in this study simulated photosynthesis recovery pattern reasonably well after combined stresses at 25 °C and 30 °C but, T sum = 50 was still too high for recovery after severe heat (35 °C) and WS. However, T sum = 20 accurately reproduced the recovery pattern and the number of days taken to fully recover from heat and drought stress at all temperature levels as observed in the measured data. The recovery from high temperature stress (T sum) function in DairyMod is also used for simulating summer dormancy in addition to the recovery of summer active species following short term heat stresses. For areas where prolonged summer droughts create accumulated soil moisture deficits above 700 mm, summer dormant species like phalaris (*Phalaris aquatica* L.) are more persistent than perennial species that lack summer dormancy [51]. In DairyMod, summer dormancy is simulated by allowing long recovery periods after heat stress (T sum = 200) so that those species spend the whole summer period without simulating any growth until next autumn where there are no more days reaching the high temperature stress threshold. However, for areas where summer droughts are not so severe, summer active species like perennial ryegrass are more productive and the high temperature stresses that occur in such areas are usually short term [52]. It has been shown that the summer active pasture species can recover such conditions [14,53]. Therefore, parameterizing this high temperature recovery function is very important for the accurate estimation of summer pasture production and when conducting climate change impact studies under future climate scenarios.

Comparison of DairyMod predicted pasture growth rates using air and leaf temperatures indicated that there was a large uncertainty in yields when the leaf temperatures were ignored particularly at the medium rainfall warm temperate climate at Dookie. Yield increase when simulating with leaf temperature ranged from 14–126%, compared to air temperatures at Dookie during late spring and summer months. This could be due to leaf temperatures getting closer to optimum for photosynthesis at higher temperatures and transpirational cooling avoiding the high temperature threshold of 30 °C in irrigated simulations. In a study comparing different sterility functions of rice models, van Oort et al. [54] reported similar observations showing that ignoring the transpirational cooling effect overestimated the spikelet sterility by 14–73%. 

While many studies have shown that the air temperature is a poor predictor in terms of plant production [55,56], most crop simulation models still using air temperature to represent canopy/leaf temperature. This could be mainly due to the complexity of using energy budget and the requirement of more detailed weather and plant (stomatal conductance) data for the energy budget estimation. In this study, energy budget for leaf was used for the estimation of leaf temperature but the most applicable component for plant population would be the canopy temperature. Canopy temperature calculation requires more information such as heat storage in the soil and canopy conductance which were not measured in this study. While further research is required to simplify the approach and find better ways to integrate leaf/canopy temperature into crop simulation models, this approach improved the simulation of perennial ryegrass under consecutive combined heat and drought stresses. 

## 4. Materials and Methods

### 4.1. Validation of Leaf Energy Budget Equation

#### 4.1.1. Experimental Description

A controlled environment experiment was conducted to collect the information required to validate leaf energy budget equation and simulation approach used in this study. Full experimental details, including the experimental design, treatments, and pasture species, were provided in [14] and only a short description is provided here. Four temperate perennial pasture species including three grasses; perennial ryegrass (*Lolium perenne* cv. Base AR37), cocksfoot (*Dactylis glomerata* cv. Savvy), tall fescue (*Festuca arundinacea* cv. Quantum II Max P) and a broad leaf; chicory (*Cichorium intybus* cv. Puna II) were grown in poly vinyl chloride tubes (height 75 cm and diameter 10 cm) inside a glasshouse in the Faculty of Veterinary and Agricultural Sciences, The University of Melbourne. Plants were well-watered and fertilised during the initial growing stage in the glass house. After eight weeks of vegetative growth, plants were transferred into three separate growth chambers and allowed to adapt to conditions for two weeks. The first week before the treatments were imposed was considered as the pre-treatment period. Plants in each chamber were then exposed to consecutive seven-day heat and water stress treatments each followed by a seven-day recovery period. Three temperature levels were allocated to three growth chambers including control = 25/15 °C, day/night, moderate heat stress = 30/20 °C, day/night and severe heat stress = 35/25 °C, day/night. At each temperature level, a group of plants were fully irrigated daily to the field capacity throughout the experiment (well-watered, WW) and irrigation was arrested during consecutive seven-day temperature treatments in the water-stressed (WS) plants. All pots were well watered during the pre-treatment and the recovery periods to the field capacity. 

Each growth chamber contained 40 plants (4 species × 5 replicates × 2 irrigation treatments) arranged in eight rows and five columns. Watering treatments (WW and WS) were allocated in alternative rows in each chamber. Species were randomised in both row- and column-wise directions.

#### 4.1.2. Measurements

Diurnal variation of temperature was implemented inside growth chambers by gradually increasing and decreasing air temperature changing between night time minimum to day time maximum over a period of 3 hours. Temperature inside the chambers were recorded every minute by a data logger in each growth chamber. Relative humidity (RH%) inside the chambers were set at 70% and light intensity was maintained at 900 µmolm^−2^s^−1^ (range of 844–1030) using high pressure sodium lamps and incandescent lights. Maximum leaf widths of 10 leaves were recorded from each pasture species and averaged to calculate leaf characteristic dimension (d = 0.72 × maximum leaf width in the direction of wind flow).

The leaf photosynthetic rate (µmol CO_2_ m^−2^ s^−1^) of the youngest fully expanded leaf was measured in perennial ryegrass three times per week throughout the experiment using Li-6400 portable gas exchange system (LI-COR Inc., Lincoln, USA) under the given lighting conditions of the chamber and CO_2_ concentration set at 400 ppm. Modulated chlorophyll fluorescence was also measured in alternative days throughout the experiment. Since the grasses have a narrow leaf, the whole leaf chamber was not covered by leaves. Therefore, every leaf inside the LI-COR leaf chamber was photographed using a digital camera (keeping equal distance between leaf chamber and camera) when taking photosynthesis measurements to compute the leaf area within the leaf chamber. The leaf area was analyzed using ImageJ software [57]. Photosynthesis was recomputed for the calculated leaf area using LI-6400 simulator 5.3.2. Stomatal water conductance and the leaf-to-air vapor pressure deficit values over day 2, 4 and 7 were also extracted from the LI-COR 6400 after recomputing the data, as shown in Figure 7. Photosynthesis data from perennial ryegrass were statistically analyzed using linear mixed models in GenStat (16^th^ edition) taking rows and columns in each chamber as the random effects and temperature, watering treatment and time as fixed effects. Three-way interaction was significant (*P* < 0.05), however to reduce the complexity of the analysis, only the means between WW and WS plants at each temperature on each day are presented in this study (Figure 3a,b).

Thermal images of 3–5 plants in each species were captured using infra-red camera (FLIR T series; model B 360) keeping the plants inside the chamber during the days 2, 4 and 7 of the second heat and WS treatment. In each thermal image, pixels of the pot and the background were eliminated by selecting the maximum and minimum temperatures within the plant canopy using a code written in MATLAB R 2014b [58]. Pixels selected within the canopy were then averaged to calculate the average leaf temperature of each plant. Air temperature values inside chambers were also recorded when capturing each thermal image using a mercury thermometer in addition to the recorded temperatures inside chambers to get the accurate air temperature reading at that point time. Average leaf temperatures of species were analyzed using two sample *t*-tests at each temperature level to test the hypothesis; H0: mean leaf temperature of WW plants = mean leaf temperature of WS plants. 

### 4.2. Leaf Temperature Calculation Using Energy Budget Equation 

Temperatures of pasture leaves were calculated using air temperature, radiation, leaf characteristic dimension [24,59], wind speed, relative humidity (RH%) and stomatal conductance using leaf energy budget for wet/humid operating systems (Chapter 14. Equation (14.6)) [24] on days 2, 4 and 7 of the second heat and WS treatment consistent with the timing of the leaf temperature measurements (using infra-red images). The leaf energy budget can be written as in Equation (1).

(1) $$Tl=T_{a}+\frac{\gamma^{*}}{s+\gamma^{*}}+\left[\frac{R_{ni}\;\;\;}{g_{Hr}Cp}-\frac{D}{P_{a}\gamma^{*}}\right]$$

where Tl is leaf temperature (°C), $T_{a}$ is air temperature (°C), $\gamma^{*}$ is apparent psychrometer constant (C^−1^), s is slope of saturation mole fraction function, $R_{ni}$ is isothermal net radiation (Wm^−2^), $g_{Hr}$ is sum of boundary layer (gHa; mol m^−2^ S^−l^) and radiative (gr; mol m^−2^ S^−1^) conductance, Cp is specific heat of air (J mol^-1^ C^-1^), D is vapor pressure deficit (kPa) and $P_{a}$ is atmospheric pressure (kPa). $R_{ni}$ was used for leaf temperature modelling because it does not depend on the leaf surface temperature. $R_{ni}$ was calculated using Equation (2).

(2) $$R_{ni}=\;SWR_{abs}+LWR_{in}-\;LWR_{out,i}$$

where $SWR_{abs}$ is absorbed short wave radiation, $LWR_{in}$ is incoming long wave radiation and $LWR_{out,i}$ is isothermal outgoing long wave radiation. 

Monocot plants (grasses) have stomata on both sides of the leaf (amphistomatous), while dicots (chicory in this study) have stomata only on one side (hypostomatous). To account for this feature, vapour conductance was calculated for grasses and chicory separately in the energy budget equation. For chicory, only the first half of Equation (3) was used.

(3) $$g_{v}=\;\frac{0.5\;g_{vs}^{ab}\;g_{va}}{g_{vs}^{ab}+g_{va}}+\frac{0.5\;g_{vs}^{ad}g_{va}}{g_{vs}^{ad}+g_{va}}$$

where $g_{v}$ is the vapor conductance, $\;g_{vs}^{ab}$ is abaxial stomatal conductance, $g_{vs}^{ad}$ is adaxial stomatal conductance and $g_{va}$ is boundary layer conductance for vapour. $g_{v}$ is used to calculate $\gamma^{*}$. Step by step calculation of the leaf temperature can be found in [24]. 

A range of statistics were calculated to test the adequacy of the leaf energy budget equation to predict the leaf temperatures of four pasture species with sufficient accuracy, based on methods reported in Tedeschi [60]. The statistics include; mean bias (the difference between measured and modelled data), the regression estimate of coefficient of determination (R^2^); Pearson’s correlation coefficient (r), mean prediction error (MPE) where <5% represents excellent model prediction, 5–10% represents very good, 10–20% represents moderate and more than 20% represents poor model prediction [61]; for the modelling efficiency (MEF), where above 0.5 is ideal and lower than zero indicates that the model predictions are worse compared to measured values; variance ratio with 1 indicating same amount of variation in measured and modelled data; bias correction factor (Cb) with 1 indicating best fit and lower than 1 indicating bias from the 1:1 reference line, and finally, the Concordance Correlation Coefficient (CCC), which is also known as reproducibility index that simultaneously account for the accuracy and precision with 1 indicating the best fit.

### 4.3. Simulation of Photosynthesis Pattern; Comparison Between Tair and Tleaf

Net positive photosynthesis rates of perennial ryegrass were simulated using the DairyMod biophysical model during consecutive heat and water stresses to compare the pattern with the measured data. Data for perennial ryegrass was used in this comparison as it is the most commonly grown pasture species in SE Australia. Six simulations were built to represent the three temperature and two watering levels separately. To run the simulations, climate files required for DairyMod were prepared using the data recorded in each growth chamber including light intensity (µmol m^−2^ s^−1^), maximum and minimum daily temperatures (°C); RH%, vapor pressure (kPa) and wind speed (used a constant value of 2 ms^−1^). One set of simulations were run using the measured climate data including the maximum daily air temperature. Another set of simulations were run substituting the maximum daily temperatures with the leaf temperatures calculated for each day using leaf energy budget equation. Maximum air temperatures inside each growth chamber and leaf temperatures for WW and WS simulations are shown in Figure 8. Canopy net positive photosynthesis values (kg C/ha.d) from DairyMod were used for the analysis. While net negative photosynthesis values were predicted by DairyMod during the combined heat and water stresses due to growth and maintenance respiration, these days were given the value of zero in this study consistent with the net positive pasture growth rates (Figure 6). Patterns of net positive photosynthesis using leaf temperature and air temperature were then compared with the measured leaf photosynthesis data. 

Since measured and modelled photosynthesis values were in different units, they were transformed to comparable scales using response ratio (RR). RR was calculated as Psis_i_ / Psis_control_ where Psis_i_ is the photosynthesis value on i^th^ day and Psis_control_ is the corresponding control value. RR gives the effect size of each temperature and watering treatment on each day compared to the WW control treatment (25 °C WW). The RR of the 25 °C, WW treatment in both measured and modelled data sets were always equal to one as the value of each day is divided by the same value. RR was calculated for both measured and modelled photosynthesis values separately and then used for the comparisons. Several regression model evaluation statistics were used, such as residual mean squared error (RMSE) and mean absolute error (MAE) to calculate the error rate of modelled data. Photosynthesis data measured only at controlled and moderate heat stress treatments were used in the analysis. Photosynthesis data measured at the severe heat stress treatment was not used for comparisons due to instrumental error occurred during measurement. However, the pattern of photosynthesis was modelled for all three temperature levels and photosynthesis pattern at severe temperature treatment (35/25 °C, day/night) was visually compared with the pattern of heat and/or drought stress responses of other growth and physiological measurements such as maximum quantum yield of photosystem II, relative leaf water content and the leaf elongation rate described in [14].

### 4.4. Parameterization of High Temperature Stress Recovery (T-sum) Function

Perennial ryegrass in the DairyMod biophysical model experience high temperature stress (high temperature stress-onset) at 30 °C and the stress become maximum (high temperature stress-full) at 35 °C. Recovery of pasture species from high temperature stress is modelled using an empirical function called T-sum recovery. The model default T sum for perennial ryegrass is 100 [34]. This means if the summation of 25 and the mean daily temperature after the high-temperature stress period reaches 100, the perennial ryegrass will fully recover from heat stress. For example, if the mean temperature of the following day is 20 °C, five heat units accumulated in that day. After 20 days of mean temperatures of 20 °C, the perennial ryegrass will fully recover from heat stress. This function was not well-parameterized in the model. The model uses arbitrary values for each species when modelling high temperature stress effects on pastures. In this study, T-sum recovery for perennial ryegrass was adjusted to match the days to recover after stress relief using the measured photosynthesis data. 

### 4.5. Evaluating Effects of Using Leaf Temperature Compared to Air Temperature on Pasture Growth Rate 

To evaluate the effects of leaf temperature on pasture production compared to air temperature, perennial ryegrass was simulated using both approaches (Tair and Tleaf) separately at two contrasting sites in south eastern (SE) Australia (high rainfall cool temperate site at Ellinbank (Lat. −38.25°, Long. 145.93°) and medium rainfall warm temperate site at Dookie (Lat. −36.37°, Long-145.70°)) spanning the period from 1960–2015. Climate data for each site (solar radiation in MJ/m^−2^, maximum and minimum temperatures in °C, rainfall in mm, evaporation in mm and RH%) were obtained from the SILO database service [62]. Soil type details of the two sites were extracted from [5]. Simulations were managed as a cutting trial where pastures were harvested to a residual level of 1.4 t DM/ha at the end of each month. Pastures were grown under nutrient nonlimiting conditions. Rainfed and irrigated pastures were simulated for each site separately. The rainfed simulation was run to capture the water limiting growth of pastures during dry months where transpirational cooling does not occur and thereby increasing leaf temperatures of plants occurs. In the irrigated simulation, irrigation was applied as required all year so that no water stress was simulated. The irrigation rule used in DairyMod was to apply 50 mm of water when the rainfall deficit (cumulative PET—rainfall) exceeded 25 mm over a five-day interval.

During simulations, the effect of transpiration on pasture growth was incorporated using modelled leaf temperature in two-step process. First, the model was run using the described climate data (obtained from SILO database) and growth limiting factor water (GLF_W) values on each day over 1960-2015 were extracted from export file for each site. GLF_W was then used to incorporate the transpirational cooling effect by adjusting stomatal conductance in the leaf energy budget, using a similar approach to a previous study [29]. GLF_W range from 0 to 1 where 1 means there is no limitation to growth while 0 means total limitation. For each GLF_W value, a corresponding stomatal conductance was allocated based on the measured values for perennial ryegrass as shown in Figure 7. Maximum stomatal conductance of 0.4 mol H_2_O m^−2^s^−1^ was allocated to GLF_W = 1 to simulate maximum transpiration and it was progressively decreased to 0.005 mol H_2_O m^−2^s^−1^, which is equal to GLF_W = 0 to simulate little or no transpiration. Stomatal conductance values allocated for a range of GLF_W are shown in Table 4. These stomatal conductance values were within the range of measured values under different soil water status and mid-day leaf water potential values for perennial ryegrass reported by [63]. As the next step, leaf temperature was calculated for each day using leaf energy budget equation using radiation, vapor pressure, wind speed, and maximum daily temperature from the climate file, and the stomatal conductance values allocated to GLF_W on each day. The calculated leaf temperature was then used as the maximum daily temperature values in the second run of the model. In the irrigated simulation, GLF_W was always equal to 1. To account for this feature, leaf temperature for irrigated plants were calculated using the maximum stomatal conductance of 0.4 mol H_2_0 m^−2^s^−1^ to represent maximum transpirational cooling. Using this approach, the environment experienced by the canopy due to transpirational cooling was incorporated into the model rather than using air temperature. The monthly pasture growth rates (kg DM/ha.day) simulated using air temperature and leaf temperature were analyzed using two sample *t*-tests for both irrigated and rainfed conditions and uncertainty was computed as percentage difference of net positive growth rates compared to air temperature.

## 5. Conclusions

The four pasture species used in this study showed limited homeothermy under WW conditions, suggesting that pastures can buffer temperature variations in a range of ambient air temperatures. A leaf energy budget equation modelled temperatures of the grasses better than chicory under different temperatures and watering levels. The leaf temperature modelled using an energy budget equation better simulated the heat stress impacts on perennial ryegrass compared to the air temperature, suggesting that the uncertainty of using air temperature can be reduced if leaf temperatures were used in crop simulation models. Weather variables available from the meteorological stations (radiation, maximum and minimum temperatures, vapor pressure, RH% and wind speed) together with crop-specific variables (leaf characteristic dimension, stomatal conductance) and the soil moisture stress index (GLF_water) allows for the approximation of leaf temperature using the leaf energy budget. When using this approach in DairyMod, simulations using leaf temperature under irrigated conditions showed increased pasture growth rates during the late spring and summer months at the Ellinbank and Dookie sites, with the highest increase predicted at the medium rainfall site at Dookie. This approach can be used to model pasture production in other temperate and Mediterranean climates of the world where high temperature stress is an ongoing problem. While further research is required to better represent the canopy temperature in crop simulation models using an energy budget approach, this study demonstrated that leaf temperature can better simulate pasture responses under consecutive combined heat and water stresses. 

## Figures and Tables

**Table 1:** Summary statistics calculated to assess the adequacy of the leaf energy budget equation in modelling leaf temperature (all data, for perennial ryegrass, cocksfoot, tall fescue, chicory, well-watered and water-stressed plants). Cb, bias correction factor; CCC, concordance correlation coefficient.

Model Statistics	All Data	Perennial Ryegrass	Cocksfoot	Tall Fescue	Chicory	Well-Watered	Water-Stressed
Mean (measured)	30.02	30.05	29.83	30.23	29.93	29.32	30.74
Mean (calculated)	31.00	30.18	30.31	30.61	32.99	30.37	31.65
Mean bias	−0.98	−0.13	−0.48	−0.39	−3.06	−1.05	−0.91
R2 (Coeff. of determination)	0.89	0.95	0.97	0.96	0.94	0.86	0.91
r (Pearson’s correlation coeff.)	0.94	0.98	0.99	0.98	0.97	0.93	0.95
Mean Prediction Error (MPE)	5.88%	3.18%	3.03%	2.86%	10.79%	6.38%	5.36%
Modelling Efficiency (MEF)	0.80	0.94	0.95	0.95	0.37	0.76	0.83
Variance Ratio (V)	0.92	0.94	0.93	0.97	0.96	0.91	0.91
Cb	1.00	0.97	1.00	1.00	1.00	1.00	1.00
CCC	0.94	0.95	0.98	0.98	0.97	0.92	0.95
n	294	76	69	79	70	150	144

**Table 2:** Correlation coefficients between measured and simulated (with air temperature versus leaf temperature and Tsum = 50 versus Tsum = 20) photosynthetic rates of perennial ryegrass at control (25 °C) and moderate heat stress (30 °C) under WW and WS treatments. Correlation coefficients (r) shown in bold are significant (*P* = 0.05).

		Tsum R = 50		Tsum R = 20	
		T Air	T Leaf	T Air	T Leaf
25 °C	WW	−0.39	−0.22	−0.39	−0.14
	WS	**0.87**	**0.86**	**0.86**	**0.86**
30 °C	WW	0.18	−0.07	0.09	−0.09
	WS	**0.88**	**0.93**	**0.86**	**0.87**

**Table 3:** Root mean square error (RMSE) and mean absolute error (MAE) comparisons for response ratios between measured data and simulated data with air temperature and leaf temperature at 25 °C WW and 30 °C, WW and WS plants.

	(RMSE)		(MAE)	
	Tair	Tleaf	Tair	Tleaf
25 °C WS	0.16	0.15	0.12	0.13
30 °C WW	0.54	0.36	0.42	0.25
30 °C WS	0.24	0.26	0.19	0.20

**Table 4:** Stomatal conductance values of perennial ryegrass used in the leaf energy budget equation when calculating leaf temperatures for each day at Dookie and Ellinbank. Stomatal conductance values used in this table came from the measured values shown in Figure 7 and they are allocated to different ranges of GLF_water values.

GLF Water Range	Stomatal Conductance mol m^−2^ s^−1^
0.91–1	0.4
0.81–0.9	0.225
0.71–0.8	0.18
0.61–0.7	0.11
0.51–0.6	0.05
0.41–0.5	0.035
0.31–0.4	0.025
0.21–0.3	0.01
0.11–0.2	0.008
0–0.1	0.005
